# Adaptive Binocular Fringe Dynamic Projection Method for High Dynamic Range Measurement

**DOI:** 10.3390/s19184023

**Published:** 2019-09-18

**Authors:** Changzhi Yu, Fang Ji, Junpeng Xue, Yajun Wang

**Affiliations:** 1 Institute of Mechanical Manufacturing Technology, China Academy of Engineering Physics, Mianyang 621999, China; yuczcaep@163.com; 2 School of Aeronautics and Astronautics, Sichuan University, Chengdu 610065, China; 3 State Key Laboratory of Information Engineering in Surveying, Mapping and Remote Sensing, Wuhan University, Wuhan 430079, China; yjwangisu@whu.edu.cn

**Keywords:** three-dimensional measurement, high dynamic range, structured light sensor, adaptive binocular fringe dynamic projection, binocular calibration

## Abstract

Three-dimensional measurement with fringe projection sensor has been commonly researched. However, the measurement accuracy and efficiency of most fringe projection sensors are still seriously affected by image saturation and the non-linear effects of the projector. In order to solve the challenge, in conjunction with the advantages of stereo vision technology and fringe projection technology, an adaptive binocular fringe dynamic projection method is proposed. The proposed method can avoid image saturation by adaptively adjusting the projection intensity. Firstly, the flowchart of the proposed method is explained. Then, an adaptive optimal projection intensity method based on multi-threshold segmentation is introduced to adjust the projection illumination. Finally, the mapping relationship of binocular saturation point and projection point is established by binocular transformation and left camera–projector mapping. Experiments demonstrate that the proposed method can achieve higher accuracy for high dynamic range measurement.

## 1. Introduction

Due to the advantages of high speed, high accuracy, and full light field, fringe projection profilometry (FPP) based on structured light sensor [1,2,3] has become the most promising three-dimensional (3D) data acquisition technique in many fields, such as quality control [4,5,6], reverse engineering [7,8], and others [9,10,11]. Generally, the system of fringe projection profilometry consists of one camera and one projector. There are three steps to obtain the 3D data of the object measured by FPP. First, the designed fringe patterns are projected onto the surface of measured object one by one and the deformed fringes are captured by the camera simultaneously. Secondly, the phase information can be calculated by the deformed fringes. Finally, the 3D point cloud of the measured object can be reconstructed accurately with the calibrated parameters of the system [1,2,3]. The 3D data can provide an effective evaluation mean for surface inspection, precision manufacturing, automatic assembly, and other fields. The literature [1,2,3,6] shows that FPP is more suitable for diffuse reflective surfaces and the surface reflectance changes little. However, when objects with high dynamic range (HDR) were measured directly by FPP, some saturated regions will appear in fringe images, which challenge the completeness of phase information and measurement accuracy.

Addressing that it is difficult to improve the dynamic range by hardware for the most camera sensors [6], many experts have carried out a series of approaches to solve the HDR measurement problem. The methods can be divided into several categories: multi-exposure method [12,13,14], light intensity adjustment method [15,16], color invariance method [17,18,19], polarization method [20,21,22], adaptive fringe pattern method [23,24,25,26,27], and others [28,29]. Considering that the dark regions and bright regions of the fringe image require different exposure times, multi-exposure method becomes a way for HDR problem [12,13,14]. Zhang [12] proposed an HDR scanning technique. The brightest but not saturated pixels were chosen from a set of fringe images with decreasing exposures to generate the new fringe images. Song [13] proposed an active reflection suppression method by multiple exposure image fusion to achieve high-precision 3D measurement. However, for the multi-exposure method, the required number of exposures and each exposure time depend seriously on human experience, and there is still a lack of quantitative way to choose an appropriate exposure time. The light intensity adjustment method is another way applied for HDR problem [15,16]. Kofman [15] projected a series of fringe patterns with decreasing maximum input gray value onto the object surface. The maximum gray value but not saturated pixels were used to synthesize the fringe images. For improving the signal-to-noise ratio for low reflective surface, Babaie [16] proposed a method to improve the dynamic range of fringe projection system to measure the objects with varying surface reflectivity. Similar to the multi-exposure method, this method needs to project a large number of different intensities, so the projection efficiency is very low. Color invariance method has also been proposed to solve the HDR measurement [17,18,19]. Benveniste and Ünsalan [17] applied color invariant method to solve the problem of scanning bright surfaces under different ambient illuminations. Chen [18] proposed a fringe pattern projection method by fusing different color patterns from multi-viewpoints. However, the accuracy of color invariance method is limited [29]. The polarization methods have been developed to handle the shiny surface problem [20,21,22]. In [24], epipolar images with speckle patterns were utilized to eliminate the effects of inter-reflections. The authors of [22] presented a specularity removal method based on polarization imaging through global energy minimization. Obviously, polarization methods require additional hardware and are time-consuming. The adaptive fringe pattern method is another solution, which computes the optimal illuminations according to the correspondence of camera–projector [23,24,25,26,27]. For the problem of strong internal reflection, Xu and Aliaga developed an adaptive corresponding algorithm [23], which may take hours to inspect an unknown scene. Li and Kofman [24] proposed an adaptive fringe pattern projection method by adapting the maximum input gray level. Lin presented a fast 3D shape measurement technique to improve the signal-to-noise ratio during the measurement [25]. Farahi [26] put forward an inverse projected-fringe technique for on-machine inspection, based on the correspondence of projector-part-camera. Zhang [27] studied a method to calculate several groups of fringe patterns with optimal light intensities generated based on the intensity response function of camera. However, when calculating the optimal light intensity, the threshold of reflectivity component must be set manually. From the perspective of projection efficiency, the adaptive fringe pattern method can achieve better results for HDR problem. 

However, most methods mentioned above belong to monocular fringe projection system, which are sensitive to image saturation and projection non-linear gamma effect. In order to improve the projection efficiency and reduce the influence of image saturation and gamma non-linear effect, combining with the advantages of binocular vision and monocular fringe projection, we propose an adaptive binocular fringe dynamic projection method by adjusting adaptively the pixel-to-pixel projection intensity. First, the flowchart of adaptive binocular fringe dynamic projection method is presented in detail. Then, an adaptive optimal projection intensity method based on multi-threshold segmentation is presented to adjust the projection illumination. Finally, mapping correspondence of binocular saturation point and projection point is established to modify the projection gray-level of saturation point. 

The organization is as follows. Section 2 introduces the basic principle of FPP. In Section 3, adaptive binocular fringe dynamic projection method is explained in detail. The experimental results are given in Section 4. Conclusions are presented in the last section.

## 2. Principle of Fringe Projection Profilometry

For higher accuracy, sinusoidal fringe pattern and phase-shifting method are adopted. For a fringe projection sensor system, numerous-step phase-shifting algorithms have been developed and applied in most researches, due to the excellent performances such as accuracy, point density, and efficiency [2,3]. The projected intensity can be represented as,

(1) $$I\left(x,y\right)=I_{A}\left(x,y\right)+I_{B}\left(x,y\right)\cos\left[\varphi \left(x,y\right)+\delta_{N}\right],\;\delta_{N}=\frac{k^{*}2\pi }{N},N=3,4,5\cdots ,k=0,1,\cdots ,N-1,$$

where $I_{A}$ is the average intensity, $I_{B}$ is the intensity modulation, φ is the phase to be solved for and *N* is the number of phase-shift steps. The phase value can be described as,

(2) $$\varphi \left(x,y\right)=-\arctan\left[\frac{\sum_{n=1}^{N}I_{n}\left(x,y\right)\sin\delta_{N}}{\sum_{n=1}^{N}I_{n}\left(x,y\right)\cos\delta_{N}}\right].$$

As shown in Equation (2), the phase value is affected by higher harmonics, which is also an important factor in the generation of phase error. Since the arctangent function is used, the phase value solved for ranges (−π,π] with 2π discontinuities. Usually, the continuous phase needs to be unwrapped for FPP by phase unwrapping algorithms [2]. Through the continuous phase obtained above, the 3D shape of object can be calculated by combining with calibration parameters discussed in [30,31]. Generally, three-step phase-shifting algorithm and four-step phase-shifting algorithm with equal phase-shifting are widely used in 3D shape measurement [2]. Considering the measurement accuracy and phase calculation amount, four-step phase-shifting algorithm is preferable for the following study. 

## 3. Adaptive Binocular Fringe Dynamic Projection Method

In order to expand the application scope of fringe projection technology, this section explains the adaptive binocular fringe dynamic projection method (ABFDP) for the problem of image saturation in HDR measurement. In order to enhance the acquisition range of point cloud and reduce fitting error [31,32], the binocular vision with fringe projection is selected in this paper with the advantages of high precision and non-gamma effect of projector.

### 3.1. Flowchart of ABFDP Method

**Figure 1:**
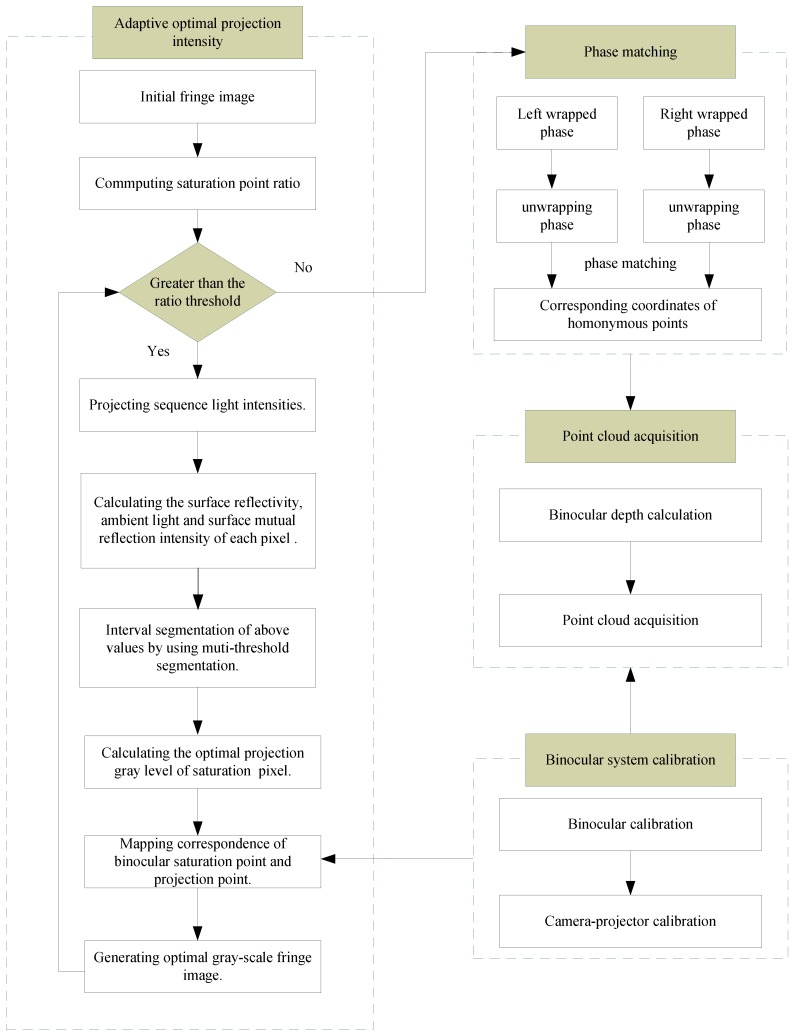
Flowchart of the adaptive binocular fringe dynamic projection method (ABFDP) method.

The flowchart of the proposed adaptive binocular fringe dynamic projection method is shown in Figure 1. The main steps are as follows.

- Step 1. Adaptive optimal projection intensity. In this step, the intensity response function and multi-threshold segmentation are used to generate the modified fringe images. Its basic principle is that the fringe images are modified iteratively by the feedback of the deformed fringe images captured by the binocular cameras.
- Step 2. Binocular system calibration. Through binocular system calibration, the mapping correspondences of binocular images and projector image are obtained.
- Step 3. Phase matching. After calculating the absolute phase, according to the principle of equal phase of homonymy point in binocular system, binocular matching points are obtained.
- Step 4. Point cloud acquisition. In this step, point cloud information of object is obtained with the principle of triangulation.

Compared with other HDR methods [23,24,25,26,27], ABFDP method extends high dynamic range measurement from monocular fringe projection to binocular fringe projection. The intensity modification mask is calculated without pre-known geometry information. The proposed ABFDP method reduces the number of fringe patterns and the number of projection iterations, which avoids complex matrix calculation and improves the projection efficiency. The ABFDP method can adaptively calculate the optimal projection intensity through multi-threshold segmentation of surface reflectivity. The adaptive projection pattern can be automatically updated for different objects. The mapping relationship of binocular saturation point and projection point is established by binocular transformation and left camera–projector mapping.

### 3.2. Adaptive Optimal Projection Intensity Method

**Figure 2:**
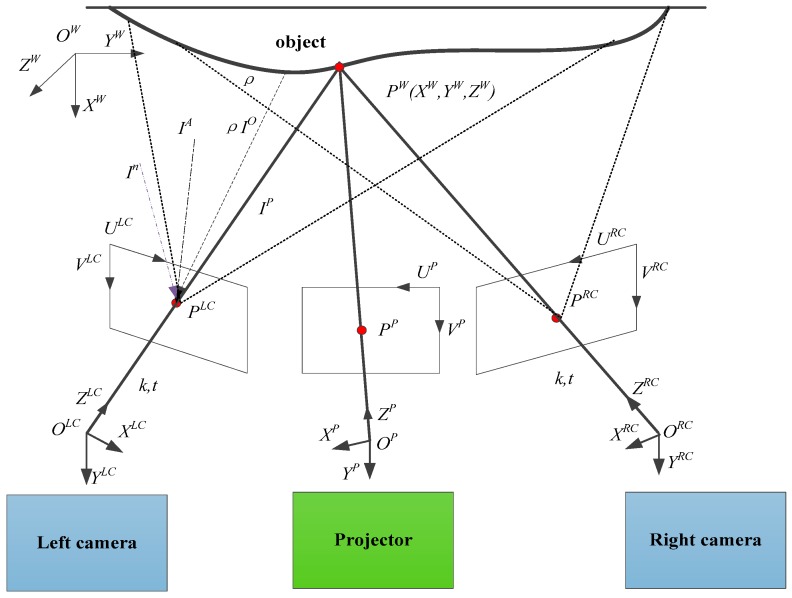
Schematic diagram of binocular fringe projection system, (1) the ambient light coming directly to the camera sensor with an intensity of $I^{A}$, (2) the projected light with an intensity of $I^{P}$, reflected by the object with surface reflectivity of ρ, $\rho I^{P}$, (3) the ambient light with an intensity of $I^{O}$, reflected by the object, $\rho I^{O}$, (4) let the camera sensitivity be k, the exposure time be t, (5) the noise of the sensor is $I^{n}$, generally obeys Gauss distribution, $I^{n}\sim N\left(0,\sigma^{2}\right)$.

In the binocular fringe projection system as shown in Figure 2, the DLP projector is used to generate sinusoidal fringe patterns and project the fringe patterns onto the object surface to code its shape information. The deformed fringes modulated by the surface of the measured object are captured by binocular cameras at the same time. The wrapped phase map and unwrapped phase map are calculated by four-step phase-shifting method. For an object point $P^{W}$ and a projector image point $P^{P}$, if two points $P^{LC}$ on left camera image corresponds to the point $P^{RC}$ on right camera image, then $\left(P^{W},P^{P},P^{LC},P^{RC}\right)$ is called homonymy point. In binocular FPP, homonymy points have the same phase values, through which the coordinates of binocular matching points can be obtained. Thereby, the object point $P^{W}$ could be calculated by stereo matching technology.

In Figure 2, $\left(O^{W},X^{W},Y^{W},Z^{W}\right)$ is the world coordinate system. $\left(O^{P},X^{P},Y^{P},Z^{P}\right)$ and $\left(U^{P},V^{P}\right)$ are the projector coordinate system and its pixel coordinate system respectively. $\left(O^{LC},X^{LC},Y^{LC},Z^{LC}\right)$ and $\left(U^{LC},V^{LC}\right)$ denote the left camera coordinate system and its pixel coordinate system respectively. $\left(O^{RC},X^{RC},Y^{RC},Z^{RC}\right)$ and $\left(U^{RC},V^{RC}\right)$ denote the right camera coordinate system and its pixel coordinate system respectively. Then, the intensity $I^{C}$ captured by each camera can be described as

(3) $$I^{C}\left(x,y\right)=kt\left\{\rho \left(x,y\right)\left[I^{P}\left(u,v\right)+I^{O}\left(x,y\right)\right]+I^{A}\right\}+I^{n},$$

where (x,y) and (u,v) denote the pixel coordinate in the camera coordinate and projector coordinate, respectively. Due to the noise of the sensor, $I^{n}$, obeys Gauss distribution, and for a given system, the camera sensitivity and the exposure time are constants, $I^{n^{\prime }}=I^{n}/kt$ also obeys Gauss distribution. Thus, Equation (3) can be rewritten as,

(4) $$I^{C}\left(x,y\right)=kt\left\{\rho \left(x,y\right)\left[I^{P}\left(u,v\right)+I^{O}\left(x,y\right)\right]+I^{A}+I^{n^{\prime }}\right\}.$$

Let

(5) $$x_{1}=ktI^{P}\left(u,v\right),\;x_{2}=kt,\;a=\rho \left(x,y\right),\;b=\rho \left(x,y\right)I^{O}\left(x,y\right)+I^{A}+I^{n^{\prime }},$$

and substituting formula (5) into Equation (4), we get,

(6) $$I^{C}\left(x,y\right)=ax_{1}+bx_{2}.$$

Assuming the reflection intensity and the ambient light on the object surface remain constant during measurement, we can estimate the values of *a* and *b* by the following way. Let $J=\sum_{i=1}^{n}{\left(I_{i}^{C}-ax_{i}{}_{1}-bx_{i2}\right)}^{2}$. In order to minimize *J*, the partial derivatives of *J* with respect to *a* and *b* can be obtained, which are all equal to zero [25].

(7) $$\{\begin{array}{c} \frac{\partial J}{\partial a}=-2\sum_{i=1}^{n}\left(I_{i}^{C}-ax_{i}{}_{1}-bx_{i2}\right)x_{i}{}_{1}=0\\ \frac{\partial J}{\partial b}=-2\sum_{i=1}^{n}\left(I_{i}^{C}-ax_{i}{}_{1}-bx_{i2}\right)x_{i2}=0 \end{array}$$

Simplification of Equation (7) is written as,

(8) $$\{\begin{array}{c} a\sum_{i=1}^{n}x_{{{}_{i}}_{1}}^{2}+b\sum_{i=1}^{n}x_{i}{}_{1}x_{i2}=\sum_{i=1}^{n}x_{i}{}_{1}I_{i}^{C}\\ a\sum_{i=1}^{n}x_{i}{}_{1}x_{i2}+b\sum_{i=1}^{n}x_{{{}_{i}}_{2}}^{2}=\sum_{i=1}^{n}x_{i}{}_{2}I_{i}^{C} \end{array}.$$

Let

(9) $$X=\left[\begin{array}{cc} x_{11} & x_{12}\\ x_{21} & x_{22}\\ \vdots & \vdots \\ x_{n1} & x_{n2} \end{array}\right],\;A=\left[\begin{array}{l} a\\ b \end{array}\right],\;I^{C}=\left[\begin{array}{l} I_{1}^{C}\\ I_{2}^{C}\\ \vdots \\ I_{N}^{C} \end{array}\right].$$

Formula (8) can be rewritten to matrix form as,

(10) $$X^{T}XA=X^{T}I^{C}.$$

From Equations (8)–(10), theoretically, two patterns are sufficient to solve a(x,y) and b(x,y). Assuming the reflectivity of the object remains constant, a set of uniform patterns with different light intensities are projected onto the measured object to increase the accuracy. If *n* patterns are used, Equation (10) can be expressed in matrix form as

(11) $$\left[\begin{array}{cc} I_{1}^{P} & kt\\ I_{2}^{P} & kt\\ \vdots & \vdots \\ I_{n}^{P} & kt \end{array}\right]\left[\begin{array}{l} a\left(x,y\right)\\ b\left(x,y\right) \end{array}\right]=\left[\begin{array}{l} I_{1}^{C}\left(x,y\right)\\ I_{2}^{C}\left(x,y\right)\\ \vdots \\ I_{n}^{C}\left(x,y\right) \end{array}\right].$$

Thus, the solution of the system of Equation (11) is

(12) $$\hat{A}=\left[\begin{array}{l} \hat{a}\\ \hat{b} \end{array}\right]={\left(X^{T}X\right)}^{-1}X^{T}I^{C}.$$

Then the surface reflectivity of each pixel is estimated to be $\hat{a}$, the ambient light and the surface mutual reflection light intensity are estimated to be $\hat{b}$, and the response function of the projector–camera can be simplified as follows,

(13) $$I^{C}\left(x,y\right)=kt\left[\hat{a}\left(x,y\right)I^{P}\left(u,v\right)+\hat{b}\left(x,y\right)\right].$$

Equation (13) is the so-called nonlinear intensity response function of projector–object–camera, which shows that for a given object and measurement scene, the gray values of the fringe image pixels captured by the cameras depend on the intensity of the projected light $I^{P}$, the camera gain *k*, and the exposure time *t*. If the camera gain and the exposure time remain constant, $I^{C}$ depends only on $I^{P}$. According to the Equation (13), optimal projection gray-level can be computed to ensure that the gray-levels of the fringe image pixels captured by the camera are in an appropriate range. The optimal projection gray-level can be calculated by the inverse function of the intensity response function as,

(14) $$I_{opt}^{P}=\frac{I_{opt}^{C}-\hat{b}kt}{\hat{a}kt}$$

Theoretically, the optimal intensity $I_{opt}^{C}$ captured by camera should avoid saturation and have high contrast. Taking into account the system noise, we have to reserve some gray level space to avoid saturation. Let $I_{opt}^{C}$ be 240 for an 8-bit camera. The corresponding optimal projection light intensity $I_{opt}^{P}$ derived from the intensity response function, can be rewritten as

(15) $$I_{opt}^{P}=\frac{240-\hat{b}kt}{\hat{a}kt}.$$

It can be seen from Equation (15) that the optimal projection gray-level of each pixel in a fringe image is different for surface with different reflectivities, i.e., the reflectivity of each pixel is not uniform, and the ambient light and surface mutual reflection intensity of each pixel are also not uniform. Equation (15) shows that each pixel will correspond to an optimal projection intensity $I_{opt}^{P}$. However, for high resolution images up to several million pixels, the computational complexity is obviously very large, which is not conducive to online measurement. From Equations (13) and (15), we notice that the projection light intensity depends on the distribution of a(x,y) and b(x,y). So, the surface reflectivity component could be divided into several intervals, and each interval corresponds to a projection light intensity. Therefore, the size of the interval length has a direct impact on the measurement result. The smaller the interval, the more light intensities will be set. This improves the measurement accuracy but sacrifices the measurement efficiency. Therefore, how to divide surface reflectivity component into several intervals is very important. Artificial experience classification of surface reflectivity in literature [27] has achieved good results. However, the way of interval division depends on manual experience, and the results are not consistent for different people. 

**Figure 3:**
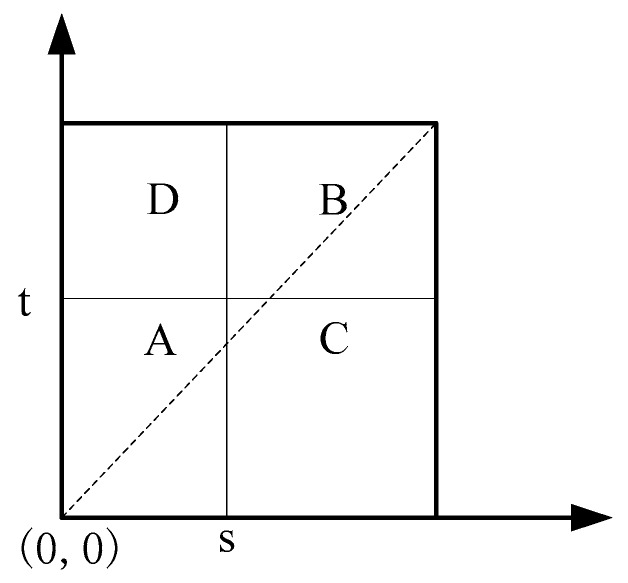
2D histogram of reflectivity, region *A* represents the target, region *B* is the background, and regions *C* and *D* are away from the diagonal.

In order to realize automatic interval segmentation, considering the surface reflectivity and the neighborhood characteristic of surface reflectivity, the 2-dimensional Otsu (2D OTSU) [33] method is introduced for threshold segmentation. As shown in Figure 3, let (s,t) denotes the thresholds, then, the 2D histogram of object reflectivity can be divided into four regions. According to the histogram, the value is close to the average value of the field at the target and background, and the difference between the value of reflectivity and the mean reflectivity value of the field at the boundary of the target and background is large. Therefore, reflectivity values in the target and background will appear around the diagonal [33]. 

According to [33], let r(x,y) be the surface reflectivity value, *d* is the width of the square neighborhood window, the neighborhood mean value of reflectivity is defined as

(16) $$n\left(x,y\right)=\frac{1}{d*d}\sum_{i=-(d-1)/2}^{(d-1)/2}\sum_{j=-(d-1)/2}^{(d-1)/2}r\left(x+i,y+j\right),$$

Define the frequency and the joint probability density of (r,n) by f(r,n) and p(r,n), the probabilities of target and background can be described respectively as,

(17) $$\begin{array}{l} \omega_{t}\left(s,t\right)=\sum_{i=0}^{s}\sum_{j=0}^{t}p_{ij}=\sum_{i=0}^{s}\sum_{j=0}^{t}\frac{f\left(i,j\right)}{M*N}\\ \omega_{b}\left(s,t\right)=\sum_{i=s+0.001}^{\max\left(r\right)}\sum_{j=t+0.001}^{\max\left(n\right)}p_{ij}=\sum_{i=s+0.001}^{\max\left(r\right)}\sum_{j=t+0.001}^{\max\left(n\right)}\frac{f\left(i,j\right)}{M*N} \end{array}.$$

To distinguish easily, an increment of 0.001 is added to the reflectivity when calculating the background probability. We can define the discrete measure matrix between the target and background as [33]

(18) $$\sigma B=\omega_{t}\left[\left(u_{0}-u_{z}\right){\left(u_{0}-u_{z}\right)}^{T}\right]+\omega_{b}\left[\left(u_{1}-u_{z}\right){\left(u_{1}-u_{z}\right)}^{T}\right].$$

Thus, the trace of discrete measure matrix, that is, the distance measure function, can be written as:

(19) $$\begin{array}{l} tr\left(s,t\right)=\omega_{t}\left[{\left(u_{0i}-u_{zi}\right)}^{2}+{\left(u_{0j}-u_{zj}\right)}^{2}\right]+\omega_{b}\left[{\left(u_{1i}-u_{zi}\right)}^{2}+{\left(u_{1j}-u_{zj}\right)}^{2}\right]\\ =\left[{\left(\omega_{t}\left(s,t\right)u_{zi}-u_{i}\left(s,t\right)\right)}^{2}+{\left(\omega_{t}\left(s,t\right)u_{zj}-u_{j}\left(s,t\right)\right)}^{2}\right]/\left[\omega_{t}\left(s,t\right)\left(1-\omega_{t}\left(s,t\right)\right)\right] \end{array}.$$

Suppose $\left(s^{*},t^{*}\right)$ represent the 2D optimal segmentation thresholds, when

(20) $$\left(s^{*},t^{*}\right)=\arg\max tr\left(s,t\right).$$

Let

(21) $$T=\left(T_{1},T_{2},T_{3},T_{4}\right)=\mathrm{Ascending}\;\left(k_{s}s^{*},k_{t}t^{*},k_{m}\left(s^{*}+t^{*}\right)/2,a_{\max}\right),$$

where $k=\left(k_{s},k_{t},k_{m}\right)$ is scale factor. The threshold T can divide the surface reflectivity into four intervals: $(0,T_{1}],(T_{1},T_{2}],(T_{2},T_{3}],(T_{3},T_{4}]$. $b_{i}$ is the maximum value of b(x,y) corresponding to each interval. The corresponding projection light intensities can be expressed as

(22) $$I_{N1}^{P}=\frac{240-b_{1}}{T_{1}},I_{N2}^{P}=\frac{240-b_{2}}{T_{2}},I_{N3}^{P}=\frac{240-b_{3}}{T_{3}},I_{N4}^{P}=\frac{240-b_{4}}{T_{4}}.$$

For the use of four-step phase-shifting algorithm, the average intensity $I_{A}$ and the intensity modulation $I_{B}$ of fringe patterns are given by

(23) $$I_{A}{}_{i}\left(x,y\right)=I_{B}{}_{i}\left(x,y\right)=\frac{I_{Ni}^{P}\left(x,y\right)}{2},i=1,2,3,4.$$

From Equations (22) and (23), groups $G_{{}_{i}}^{N}$ for four-step phase-shifting of fringe patterns are generated as follows:

(24) $$G_{i}^{4}:\;\;\left\{ \begin{array}{l} I_{4i1}^{P}=\frac{I_{4i}^{P}}{2}+\frac{I_{4i}^{P}}{2}\cos\left[\varphi \left(u,v\right)\right]\\ I_{4i2}^{P}=\frac{I_{4i}^{P}}{2}+\frac{I_{4i}^{P}}{2}\cos\left[\varphi \left(u,v\right)+\frac{\pi }{2}\right]\\ I_{4i3}^{P}=\frac{I_{4i}^{P}}{2}+\frac{I_{4i}^{P}}{2}\cos\left[\varphi \left(u,v\right)+\pi \right]\\ I_{4i4}^{P}=\frac{I_{4i}^{P}}{2}+\frac{I_{4i}^{P}}{2}\cos\left[\varphi \left(u,v\right)+\frac{3\pi }{2}\right] \end{array}\right.,i=1,2,3,4.$$

### 3.3. Mapping Correspondence of Binocular Saturation Point and Projection Point

In Section 3.2, the adaptive optimal projection intensity method solves the magnitude of the projected intensity. This subsection will answer where the appropriate projection intensities should be located, through mapping its image coordinates to the projector image coordinate system.

According to the working principle of binocular fringe projection system [34,35], stereoscopic vision calibration should be carried out, that is, to get the internal and external parameters of the left and right cameras, as well as the rotation and translation relations between the two cameras. Usually, the camera calibration principle is based on the aperture imaging model. For the left camera [35], we have

(25) $$s_{L}\left[\begin{array}{c} u_{L}\\ v_{L}\\ 1 \end{array}\right]=A_{L}\left[\begin{array}{cc} R_{L} & T_{L} \end{array}\right]\left[\begin{array}{c} x_{W}\\ y_{W}\\ z_{W}\\ 1 \end{array}\right]=M_{L}\left[\begin{array}{c} x_{W}\\ y_{W}\\ z_{W}\\ 1 \end{array}\right],$$

where ${\left(x_{W},y_{W},z_{W},1\right)}^{T}$ represents the world coordinates of any space point, ${\left(u_{L},v_{L},1\right)}^{T}$ is the pixel coordinate of the point in the left camera image, $A_{L}$ is the internal parameter of the left camera and $R_{L}$, $T_{L}$ are the rotation matrix and translation matrix of the left camera respectively, $M_{L}$ is the projection matrix of the left camera.

Similarly, for the right camera, we have

(26) $$s_{R}\left[\begin{array}{c} u_{R}\\ v_{R}\\ 1 \end{array}\right]=A_{R}\left[\begin{array}{cc} R_{R} & T_{R} \end{array}\right]\left[\begin{array}{c} x_{W}\\ y_{W}\\ z_{W}\\ 1 \end{array}\right]=M_{R}\left[\begin{array}{c} x_{W}\\ y_{W}\\ z_{W}\\ 1 \end{array}\right],$$

where ${\left(u_{R},v_{R},1\right)}^{T}$ is the pixel coordinate of the point in the right camera image, $A_{R}$ is the internal parameter of the right camera, $R_{R}$, $T_{R}$ are the rotation matrix and translation matrix of the right camera respectively, and $M_{R}$ is the projection matrix of the right camera.

External parameters of the two cameras can be obtained by calibration of monocular cameras $\left[\begin{array}{cc} R_{L} & T_{L} \end{array}\right]$, $\left[\begin{array}{cc} R_{R} & T_{R} \end{array}\right]$ and internal parameters $A_{L}$, $A_{R}$. Assuming that the projection points of a space point $P^{W}$ on the imaging plane of the left and right cameras are $P_{R}$, $P_{L}$ respectively, a coordinate system can be established [35]

(27) $$\{\begin{array}{c} P_{L}=R_{L}P^{W}+T_{L}\\ P_{R}=R_{R}P^{W}+T_{R} \end{array}.$$

Suppose that the rotation and translation matrix between two cameras is $\left[\begin{array}{cc} R & T \end{array}\right]$, thus:

(28) $$\begin{array}{l} P_{L}=R_{L}R_{R}^{-1}P_{R}-R_{L}R_{R}^{-1}T_{R}+T_{L}=\left[\begin{array}{cc} R & T \end{array}\right]P_{R}\\ R=R_{L}R_{R}^{-1},T=T_{L}-RT_{R} \end{array}$$

The above is the calibration process of binocular cameras, so that the conversion relationship between right camera and left camera can be obtained. For the calibration of DLP projector, it is usually assumed that the projector is a reverse camera, and the left camera and the DLP projector constitute a binocular system. The camera and the projector can also be calibrated through the above process, so that the internal and external parameters of the camera and the DLP projector and their correlation can also be obtained. The pixel coordinate of a point $m_{P}\left(u_{P},v_{P}\right)$ on the image plane of projector corresponds to the point $\left(x_{W},y_{W},z_{W}\right)$ in the world coordinate, while corresponding to the point $m_{L}\left(u_{L},v_{L}\right)$ of the left camera,

(29) $$\left[\begin{array}{c} u_{P}\\ v_{P}\\ 1 \end{array}\right]=A_{P}\left[\begin{array}{cc} R_{P} & T_{P} \end{array}\right]\left[\begin{array}{c} x_{W}\\ y_{W}\\ z_{W}\\ 1 \end{array}\right]$$

where $A_{P}$ is the internal parameter of projector, $R_{P}$ is a rotation matrix, $T_{P}$ is a translation matrix.

According to formula (25), the mapping relationship between image point coordinates of left camera image and projection image point coordinates can be obtained as:

(30) $$\left[\begin{array}{c} u_{P}\\ v_{P}\\ 1 \end{array}\right]=A_{P}\left[\begin{array}{cc} R_{P} & T_{P} \end{array}\right]M_{L}^{-1}\left[\begin{array}{c} u_{L}\\ v_{L}\\ 1 \end{array}\right].$$

The internal and external parameters of the binocular cameras can be obtained by Equations (25) and (26). The transformation relationship between the right camera and the left camera can be obtained by Equation (28), and the mapping relationship between the left camera and the projector can be obtained by Equation (30). Furthermore, for the saturated points of the left camera image, the corresponding coordinates of the projected image points can be obtained by mapping. For the saturated points of the right camera image, the coordinates of the projection image points can be transformed into the coordinates of the left camera image by Equation (28), and then the coordinates of the projection image points can be obtained by Equation (30). Therefore, the adaptive projection gray level correction is carried out. 

In summary, the calculation process of the proposed method is as follows,

- Step 1. Binocular camera and left camera–projector calibration. This step is mainly used to obtain the internal and external parameters of the cameras and projector, as well as the conversion relationship of the cameras and projector.
- Step 2. Projecting a set of light intensity onto the surface of object, we can get the surface reflection characteristics and the optimal projection grays.
- Step 3. Four-step phase-shifting images are collected simultaneously by left and right cameras. The saturation points in binocular cameras are identified, and then the corresponding points in the projection image are obtained according to step 1.
- Step 4. The corrected fringe images are projected onto the surface of the object to calculate the absolute phase. Therefore, the diameter results of objects can be obtained by point cloud fitting.

## 4. Experiments and Results

**Figure 4:**
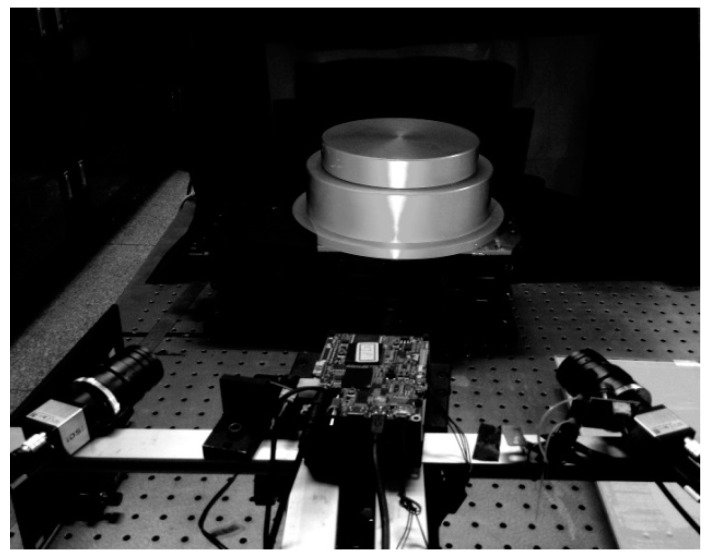
Experimental setup for binocular vision system.

In this section, we try to further evaluate the proposed method. All simulations listed here are implemented in Matlab R2018b on a laptop equipped with 2.50 GHz CPU and 4G RAM memory. To verify the performance of our method, we built a fringe projection system consists of a digital light processing (DLP) projector (model PDC03, Fuzhou Giant Vinda Photoelecyric Technology CO. LTD. China) with a projection speed of 30 fps and 1280 × 800 pixels, and two industrial CMOS cameras (model: IDS UI-3370CP-M-GL, produced by the company of IDS Imaging Development Systems GmbH in Obersulm, Germany). The cameras have a resolution of 2048 × 2048 pixels at a frame rate up to 80 fps. The fitting of point cloud data obtained to calculate the diameters in this experiment is processed by Geomagic Studio® 2013 which made by Geomagic, Inc., USA. A photograph of the experiment system is shown in Figure 4. This system was calibrated before the experiment. Experiments were conducted to verify the validity of the proposed method. 

### 4.1. Mixed Reflectivity Materials Experiment

**Figure 5:**
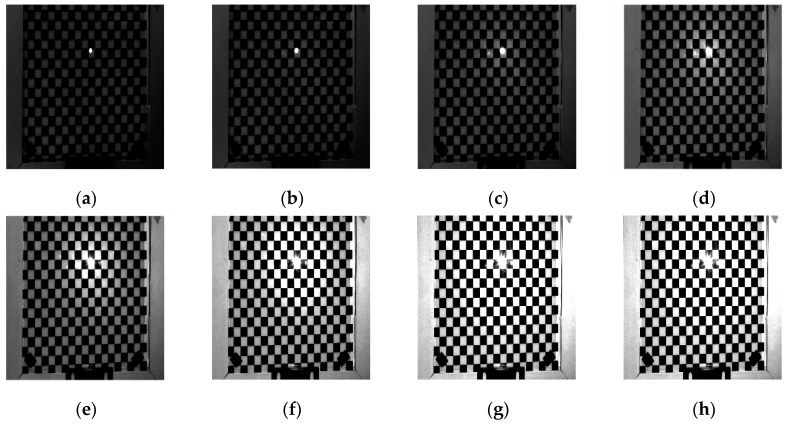
Checkerboard images captured by the right camera with different projection illumination. (**a**) With intensity of 0, (**b**) with intensity of 20, (**c**) with intensity of 40, (**d**) with intensity of 60, (**e**) with intensity of 80, (**f**) with intensity of 100, (**g**) with intensity of 120, (**h**) with intensity of 125.

**Figure 6:**
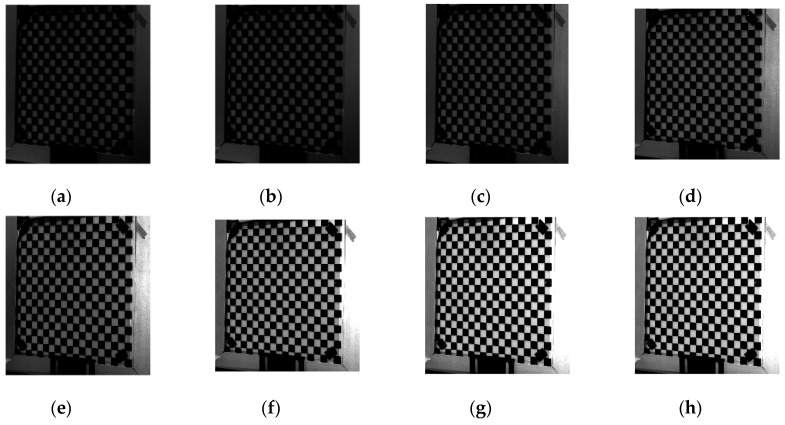
Checkerboard images captured by the left camera with different projection illumination. (**a**) With intensity of 0, (**b**) with intensity of 20, (**c**) with intensity of 40, (**d**) with intensity of 60, (**e**) with intensity of 80, (**f**) with intensity of 100, (**g**) with intensity of 120, (**h**) with intensity of 125.

**Figure 7:**
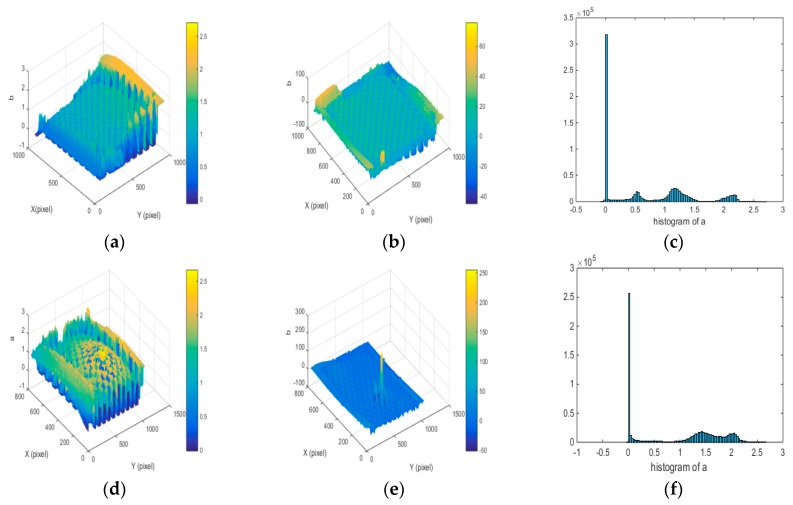
Results and histograms. (**a**) Surface reflectance *a* with right camera, (**b**) *b* with right camera, (**c**) histogram of *a* with right camera, (**d**) surface reflectance *a* with left camera, (**e**) *b* with left camera, (**f**) histogram of *a* with left camera.

In order to verify the effectiveness of the adaptive optimal projection intensity method, the glass checkerboard with a frame of aluminum alloy material is chosen as one sample for verification. As shown in Figure 5, Figure 6 and Figure 7, the surrounded frame has higher reflectivity characteristic than the glass checkerboard. The uniform light intensities are projected to the chessboard, and then the corresponding images are captured. It can be seen that with the increase of projected light intensity, the image contrast shows an enhanced trend.

It can be seen from Figure 5, Figure 6 and Figure 7 that the reflectivity characteristic of aluminum alloy material is obviously stronger than that of checkerboard. From Figure 5 and Figure 7e, an area in the image captured by the left camera is always in the state of direct reflection. Even if the light intensity is very small, this area is always in saturation state. From Figure 7, the reflectivity of the white area of checkerboard is obviously higher than that of the black area, while black areas have high ambient light intensity. As shown in Figure 7, if Zhang’s method [26] is used, surface reflectivity can be divided into three intervals: (0, 1], (1, 2], and (2, 2.7]. The corresponding projected light intensities are

$$I_{1}^{P}=\frac{240-70}{1}=140,I_{2}^{P}=\frac{240-70}{2}=70,I_{3}^{P}=\frac{240-70}{2.7}=56.$$

If our proposed method is used, surface reflectivity of left camera image is divided into three intervals: (0, 1], (1, 1.7], (1.7, 2.2], and (2.2, 2.7], and surface reflectivity of right camera image is divided into three intervals: (0, 1], (1, 1.6], (1.6, 2.3], and (2.3, 2.7]. The corresponding projected light intensities are

$$I_{L1}^{P}=\frac{240-12}{1}=228,I_{L2}^{P}=\frac{240-18}{1.7}=130,I_{L3}^{P}=\frac{240-34}{2.2}=94,I_{L4}^{P}=\frac{240-91}{2.7}=55,$$

$$I_{R1}^{P}=\frac{240-10}{1}=230,I_{R2}^{P}=\frac{240-22}{1.6}=136,I_{R3}^{P}=\frac{240-41}{2.3}=86,I_{R4}^{P}=\frac{240-73}{2.7}=62.$$

Compared with Zhang’s method, the adaptive optimal projection intensity method has better adaptability to saturated region and can fine-tune the gray-scale according to the reflectivity characteristics of objects.

### 4.2. Shiny Metal Objects Experiment

**Figure 8:**
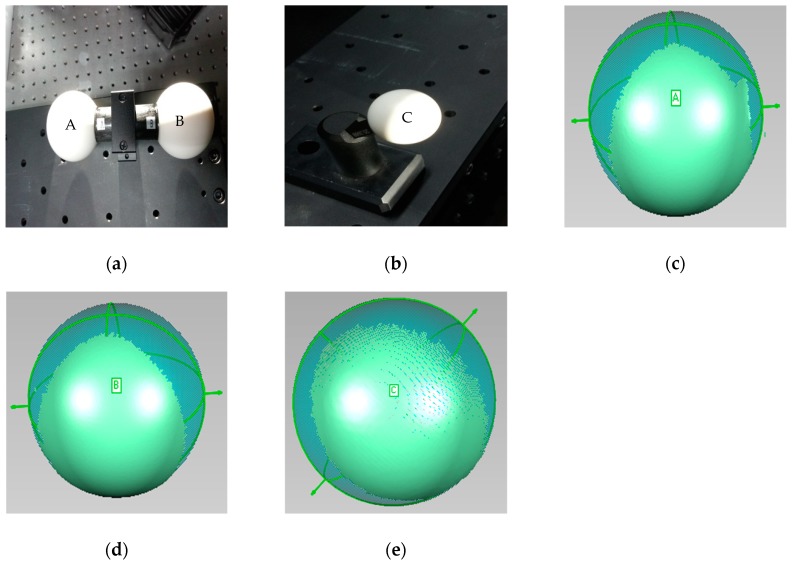
Standard calibration balls. (**a**) A and B balls, (**b**) C ball, (**c**) point cloud of A, (**d**) point cloud of B, (**e**) point cloud of C.

The precision of the fringe projection system is verified before measuring the metal objects with high dynamic reflectivity surface. Three calibration balls were designed, as shown in Figure 8, and their diameters are needed to be measured. In order to better evaluate the system accuracy, Coordinate Measuring Machine (CMM) measurement values are chosen as the actual values. In the commercial software Geomagic Studio, the diameter results of calibrated balls can be obtained by spherical fitting of point cloud information, shown in Table 1.

As can be seen from Table 1, compared with the measurement value of CMM, the mean measurement error values of the system for three calibration balls are 0.0055 mm, 0.0051 mm, and 0.0041 mm respectively, which means the fringe projection system has a higher measurement accuracy.

**Figure 9:**
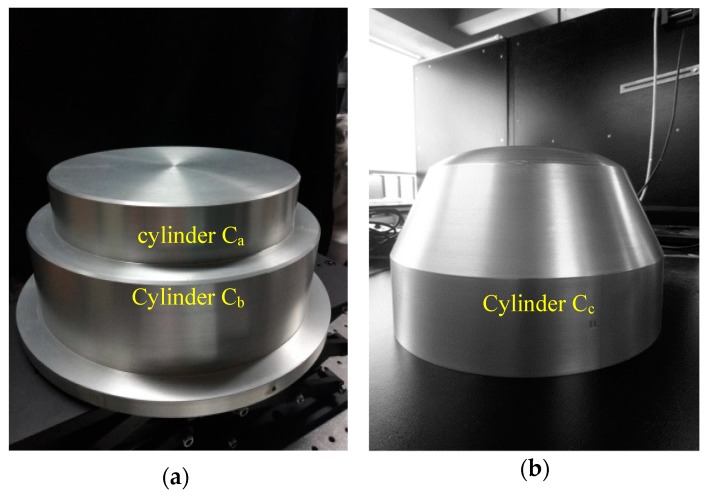
HDR objects. (**a**) Stepped cylinder object with cylinder Ca and Cb; (**b**) cylindrical shell object with cylinder Cc.

In industrial field, due to the influence of material and surface roughness, metal objects have high reflectivity characteristic, which limits the application of non-contact measurement methods. In order to verify the measuring effect of the proposed method on metal workpiece, the following two aluminum workpieces were designed, as shown in Figure 9. The measurement requirement is to measure the diameters of stepped cylinder object with cylinder C_a_ and C_b_, and cylindrical shell object with cylinder C_c_. Similar to the calibration balls, the diameter results of stepped cylinders can also be obtained by cylindrical fitting of point cloud information.

**Figure 10:**
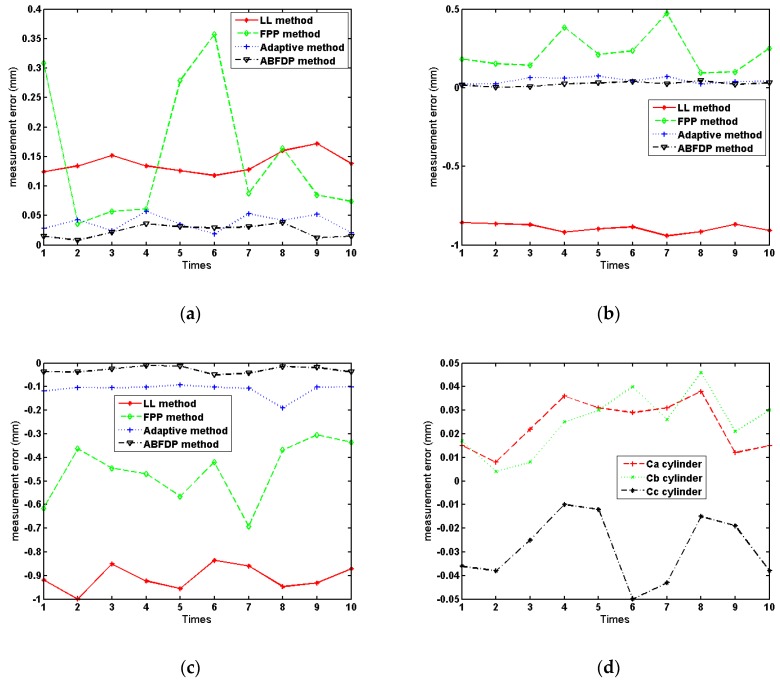
Measurement error distribution: (**a**) measurement error for cylinder C_a_; (**b**) measurement error for cylinder C_b_; (**c**) measurement error for cylinder C_c_; (**d**) measurement error using ABFDP method.

For the requirements of diameter measurement of HDR object, the measurement value with CMM is taken as the actual value. In order to verify the algorithm, the proposed method is compared with line laser method (LL method, model: LMI Gocator 2430, resolution X: 0.037mm, resolution Z: 0.006mm, points 1940), traditional fringe projection method (FPP method) and adaptive adjustment method (adaptive method), as shown in Table 2, Table 3 and Table 4. In order to observe the measurement accuracy and repetitive measurement accuracy, 10 times measurements were carried out with different methods, and the Mean value (MV), Standard deviation (STD), Root mean square error (RMSE), and Mean absolute error (MAE) were calculated as the evaluating indicators. For the convenience of visual comparison, the measured values in Table 2, Table 3 and Table 4 are displayed graphically in Figure 10.

**Figure 11:**
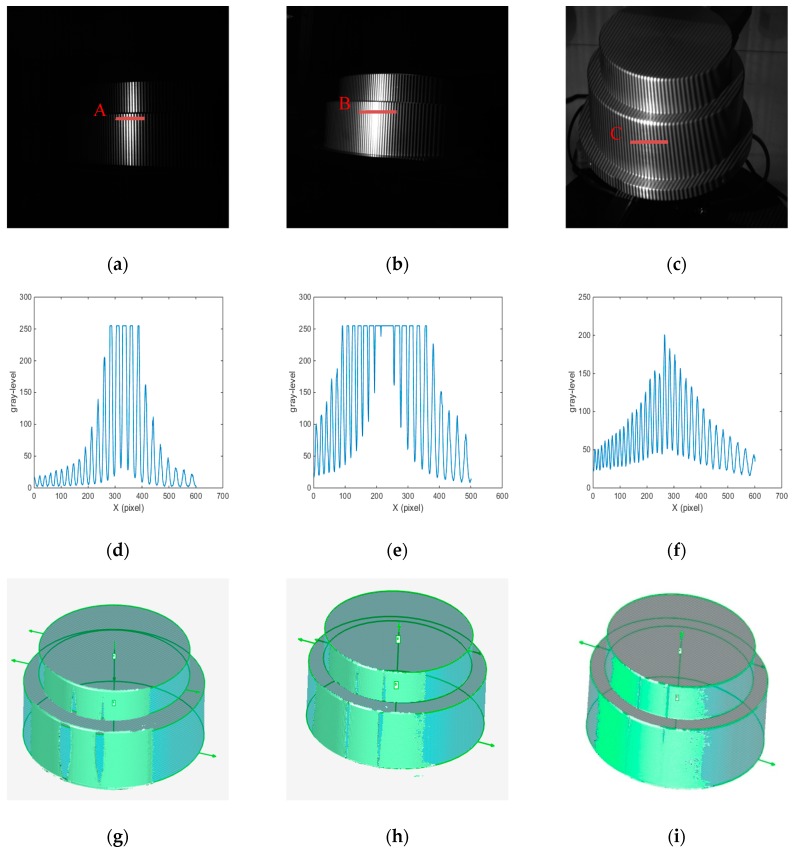
Measurement effect of stepped cylinder object. (**a**) Fringe image with FPP method, (**b**) fringe image with adaptive method, (**c**) vertical fringe image with ABFDP method, (**d**) fringe curve of A, (**e**) fringe curve of B, (**f**) fringe curve of C, (**g**) point cloud of cylinders with FPP method, (**h**) point cloud of cylinders with adaptive method, (**i**) point cloud of cylinders with ABFDP method.

The fringe patterns of cylinders C_a_ and C_b_ are shown in Figure 11. From Figure 11a,d, in order to reduce the intensity of the highlighted area, a lower projection gray level was used for the fringe image by traditional FPP method. Although the local highlighted area can be reduced, the image contrast is low, and most gray-levels are in the projection non-linear area below 50. In Figure 11b,e, compared with the traditional FPP method, the fringe contrast of adaptive method is obviously improved, but there is still a large range of bright areas in local area, which belongs to specular reflection. As shown in Figure 11c,f, the fringe contrast of ABFDP method is obviously higher than traditional FPP and adaptive method. Although some fringes are saturated locally, most gray-levels are in the projection linear region from 50 to 200. The point cloud of the cylinder is obtained by phase-shifting algorithm, and the point cloud data is imported into software of Geomagic studio for cylindrical fitting, and the fitting effect is obtained, as shown in Figure 11g–i. 

From Table 2, Table 3 and Table 4, it can be seen that different measurement methods are affected by the problem of metal surface. The mean absolute error values of line laser method are 0.139 mm, −0.892 mm, and −0.909 mm. The reason for the low detection accuracy is that the line laser method only collects the point cloud of the shorter arc, and the maximum number of point cloud is 1940. It belongs to circle fitting or ellipse fitting based on short arc, which is well known that there is a high error magnification problem in fitting short arc [32,36]. At the same time, for metal high-brightness object, the line laser stripe is still over-saturated, which easily leads to the reduction of the accuracy of stripe center recognition and affects the accurate extraction of point clouds. From Table 2, Table 3 and Table 4 and Figure 10 and Figure 11, the bright metal surface has a great influence on the traditional FPP method, which leads to the over-saturation of the image and the lack of point clouds in large areas. Meanwhile, the measurement error fluctuates greatly makes it difficult to get the real value of the object. Although the point cloud information of the traditional fringe projection method is much better than that of the line laser method, due to the influence of the metal surface, the local some areas are over-saturated, which leads to the inability of collecting the point cloud information. When these point clouds are used for circle fitting, they still belong to the problem of piecewise short arc fitting. Compared with the above two methods, adaptive method can achieve more point cloud information for high-brightness surfaces, in which MAE values are 0.037 mm, 0.047 mm, and −0.113 mm. Besides, the point cloud information collected by the adaptive method is better than that by the traditional FPP method. However, due to the influence of the bright metal surface, there are still some areas where the point cloud information cannot be extracted. Compared with the other three methods, the ABFDP method proposed in this paper effectively reduces the impact of HDR reflections, and the measurement results are closer to the actual value for the three cylinders. The mean values of the ABFDP method are 199.774 mm, 239.766 mm, and 276.270 mm and the RMSE values are 0.026 mm, 0.028 mm, and 0.032 mm, also the MAE values are 0.023 mm, 0.025 mm, and −0.029 mm. From Figure 10 and Figure 11, compared with the other three methods, the mean absolute error and fluctuation of ABFDP method are smaller, and it is closer to the actual value. At the same time, it can be seen from the Figure 10d that with the increase of the diameter of objects, the mean absolute error of this method will raise. The reason is that with the increase of the object size, the arc corresponding to the detected point cloud becomes smaller, which leads to the increase of the errors after the fitting of point cloud. As can be seen from Figure 11, the point cloud integrity of the proposed method is better than that of the traditional FPP method and the adaptive method. However, there are still some areas present the phenomenon of losing point cloud. The areas are directly opposite to the left and right cameras, and will be saturated by the camera even if the light intensity is low. The above experiments have demonstrated that ABFDP method can get highly accurate 3D measurement results. 

As stated above, we can see that the surface reflection characteristics and the size of the object will all affect the final detection accuracy when using the fringe projection method for diameter measurement. Compared with several measurement methods, the ABFDP method has the best overall performance for HDR measurement.

## 5. Conclusions

In this study, in order to solve the HDR measurement problem, an adaptive binocular fringe dynamic projection method was proposed to reduce the number of fringe patterns, which also avoids the complex matrix calculation. A novel adaptive optimal projection intensity method based on multi-threshold segmentation was established to reduce the projection gray-level according to the reflectivity information of saturated points. The mapping relationship between binocular saturation points and projection points was obtained by binocular calibration and camera–projection transformation relationship. Experimental results demonstrated that the proposed ABFDP method has the ability to precisely measure HDR objects. 

The proposed method also shares some limitations, similar to most 3D reconstruction methods based on FPP. When the objects near specular reflection, the captured fringe images are always saturated even though a little light intensity, which should be addressed in the future. 

## Figures and Tables

**Table 1:** Measurement results of calibration balls.

Calibration Balls	CMM/mm	Mean Value by ABFDP/mm	Mean Error by ABFDP/mm
A	50.7991	50.8046	0.0055
B	50.7970	50.8021	0.0051
C	253988	25.4039	0.0041

**Table 2:** Diameter measurement results of C_a_ with different methods.

Cylinder Measured	CMM/mm	Line Laser Method/mm	FPP Method/mm	Adaptive Method/mm	ABFDP Method/mm
C_a_	199.750	199.874	200.059	199.778	199.765
		199.884	199.786	199.793	199.758
		199.902	199.807	199.774	199.772
		199.884	199.811	199.807	199.786
		199.876	200.029	199.785	199.781
		199.868	200.107	199.769	199.779
		199.878	199.838	199.803	199.781
		199.910	199.914	199.792	199.788
		199.922	199.835	199.802	199.762
		199.888	199.824	199.771	199.765
MV/mm		199.889	199.901	199.787	199.774
STD/mm		0.017	0.119	0.014	0.011
RMSE/mm		0.140	0.189	0.040	0.026
MAE/mm		0.139	0.151	0.037	0.023

**Table 3:** Diameter measurement results of C_b_ with different methods.

Cylinder Measured	CMM/mm	Line Laser Method/mm	FPP Method/mm	Adaptive Method/mm	ABFDP Method/mm
C_b_	239.741	238.884	239.924	239.764	239.758
		238.877	239.893	239.766	239.745
		238.871	239.885	239.805	239.749
		238.823	240.126	239.803	239.766
		238.844	239.953	239.814	239.771
		238.856	239.977	239.785	239.781
		238.800	240.215	239.811	239.767
		238.826	239.836	239.765	239.787
		238.872	239.841	239.781	239.762
		238.834	239.993	239.782	239.771
MV/mm		238.849	239.964	239.788	239.766
STD/mm		0.027	0.122	0.019	0.013
RMSE/mm		0.893	0.252	0.050	0.028
MAE/mm		−0.892	0.223	0.047	0.025

**Table 4:** Diameter measurement results of C_c_ with different methods.

Cylinder Measured	CMM/mm	Line Laser Method/mm	FPP Method/mm	Adaptive Method/mm	ABFDP Method/mm
C_c_	276.299	275.380	275.684	276.180	276.263
		275.300	275.936	276.195	276.261
		275.448	275.853	276.194	276.274
		275.376	275.830	276.197	276.289
		275.344	275.734	276.206	276.287
		275.464	275.879	276.197	276.249
		275.440	275.607	276.192	276.256
		275.352	275.931	276.108	276.284
		275.368	275.994	276.197	276.280
		275.428	275.963	276.198	276.261
MV/mm		275.390	275.841	276.186	276.270
STD/mm		0.053	0.128	0.028	0.014
RMSE/mm		0.910	0.474	0.116	0.032
RMSE/mm		−0.909	−0.458	−0.113	−0.029
